# O034. Type of pain and onabotulinumtoxin-A in chronic migraine: four years of follow-up

**DOI:** 10.1186/1129-2377-16-S1-A89

**Published:** 2015-09-28

**Authors:** Chiara Cesaretti, Elisabetta Molesti, Francesco Lolli, Aldo Amantini, Silvia Lori

**Affiliations:** Department NeuroMuscolar Scheletric and Sensory Organs, SOD Neurophysiology, Florence, Italy; Regional Reference Center of Chemical Denervation (RRCCD), Careggi Hospital, Florence, Italy; University of Florence, Florence, Italy

## Background

Refractory chronic migraine (rCM)[1] is a debilitating neurological disorder, characterized by headache on ≥ 15 days per month for > 3 months, resistant to conventional symptomatic and/or prophylactic polytherapy. The effectiveness of the OnabotulinumtoxinA (OnabotA) was demonstrated in PREEMPT trials and approved in 2010 for CM treatment[2, 3]. The prophylactic pharmacological actions of OnabotA include: a direct antinociceptive-analgesic effect for primary peripheral afferent terminals by inhibiting release of nociceptive mediators (glutamate, substance P, CGRP)[4] and an indirect effect presumed to involve inhibition of peripheral and central sensitization in trigeminovascular neurons. The purpose of this study was to evaluate the efficacy, safety and tolerability of OnabotA as a prophylactic therapy in patients with rCM and observe the influence of the type of pain on the effectiveness of the treatment itself.

## Materials and methods

We analyzed 76 patients (64 F), mean age 52 years (23-82 yr) with rCM referred to the RRCCD of the Careggi Hospital, between 2011-2014. The patients were treated (after informed consent) with OnabotA injection in 31/39 sites at the total dosage of 155/195U every 3 months, according to the PREEMPT protocol. The frequency of headache days (F), intensity of pain (I) and the consumption of drugs (D) were measured using a Headache Diary. Two groups of patient were identified by the type of pain reported: type 1 (severe unilateral) and type 2 (moderate bilateral)[5]. For statistical analysis we used ANOVA for repeated measures and the T-test.

## Results

All 76 patients received the toxin treatment at least four times (a one-year follow-up). Of these 44/76 responded to treatment (38 F). The parameters F, I, D showed a progressive and gradual decline with time (p 0.001 for each variable), reaching the maximum effect from the fourth treatment. The F and D were statistically significantly reduced in both groups, more in patients with type 1 pain (p < 0.001), while the I group was found to ameliorate without a difference between the different pain type groups. There were no changes for age, sex and menstrual cycle. Only one patient (1.3%) dropped out of the study because of neck pain.

## Conclusions

In our four years of follow-up study a high percentage of rCM patients (58%) showed an improvement in the quality of life with a reduction of F, I and D. The kind of pain in migraine patients affects the efficacy of the treatment itself, and is more successful in patients with type 1 pain in respect to patients with type 2 pain. OnabotA is a safe treatment, well tolerated and effective as a prophylactic treatment in rCM.

Written informed consent to publication was obtained from the patient(s).
